# Association of the Salivary Microbiome With Animal Contact During Early Life and Stress-Induced Immune Activation in Healthy Participants

**DOI:** 10.3389/fpsyt.2020.00353

**Published:** 2020-05-07

**Authors:** Dominik Langgartner, Cristian A. Zambrano, Jared D. Heinze, Christopher E. Stamper, Till S. Böbel, Sascha B. Hackl, Marc N. Jarczok, Nicolas Rohleder, Graham A. Rook, Harald Gündel, Christiane Waller, Christopher A. Lowry, Stefan O. Reber

**Affiliations:** ^1^Laboratory for Molecular Psychosomatics, Department of Psychosomatic Medicine and Psychotherapy, University of Ulm, Ulm, Germany; ^2^Department of Integrative Physiology, University of Colorado Boulder, Boulder, CO, United States; ^3^Department of Psychosomatic Medicine and Psychotherapy, University of Ulm, Ulm, Germany; ^4^Department of Psychology, Friedrich-Alexander University, Erlangen, Germany; ^5^Center for Clinical Microbiology, University College London (UCL), London, United Kingdom; ^6^Center for Neuroscience and Center for Microbial Exploration, University of Colorado Boulder, Boulder, CO, United States; ^7^Department of Physical Medicine and Rehabilitation and Center for Neuroscience, University of Colorado Anschutz Medical Campus, Aurora, CO, United States; ^8^Veterans Health Administration, Rocky Mountain Mental Illness Research Education and Clinical Center (MIRECC), The Rocky Mountain Regional Medical Center (RMRMC), Aurora, CO, United States; ^9^Military and Veteran Microbiome: Consortium for Research and Education (MVM-CoRE), Aurora, CO, United States; ^10^inVIVO Planetary Health, Worldwide Universities Network (WUN), West New York, NJ, United States

**Keywords:** alpha diversity, animal contact, beta diversity, interleukin (IL)-6, salivary microbiome, Trier Social Stress Test (TSST), rural upbringing, urban upbringing

## Abstract

The prevalence of stress-associated somatic and psychiatric disorders is increased in environments offering a narrow relative to a wide range of microbial exposure. Moreover, different animal and human studies suggest that an overreactive immune system not only accompanies stress-associated disorders, but might even be causally involved in their pathogenesis. In support of this hypothesis, we recently showed that urban upbringing in the absence of daily contact with pets, compared to rural upbringing in the presence of daily contact with farm animals, is associated with a more pronounced immune activation following acute psychosocial stressor exposure induced by the Trier Social Stress Test (TSST). Here we employed 16S rRNA gene sequencing to test whether this difference in TSST-induced immune activation between urban upbringing in the absence of daily contact with pets (*n* = 20) compared with rural upbringing in the presence of daily contact with farm animals (*n* = 20) is associated with differences in the composition of the salivary microbiome. Although we did not detect any differences in alpha or beta diversity measures of the salivary microbiome between the two experimental groups, statistical analysis revealed that the salivary microbial beta diversity was significantly higher in participants with absolutely no animal contact (*n* = 5, urban participants) until the age of 15 compared to all other participants (*n* = 35) reporting either daily contact with farm animals (*n* = 20, rural participants) or occasional pet contact (*n* = 15, urban participants). Interestingly, when comparing these urban participants with absolutely no pet contact to the remaining urban participants with occasional pet contact, the former also displayed a significantly higher immune, but not hypothalamic-pituitary-adrenal (HPA) axis or sympathetic nervous system (SNS) activation, following TSST exposure. In summary, we conclude that only urban upbringing with absolutely no animal contact had long-lasting effects on the composition of the salivary microbiome and potentiates the negative consequences of urban upbringing on stress-induced immune activation.

## Introduction

Stress-related somatic and psychiatric disorders have been increasing in Western societies throughout the last decades (1, 2), with urban areas being more affected than rural ones (3, 4). Although the underlying mechanisms are not fully understood, recent studies promote the hypothesis that a compromised immunoregulatory capacity, due to diminished contact to microorganisms (i.e., “Old Friends”) with which humans coevolved, might at least in part underlie the increased disease vulnerability of individuals living in urban compared with rural areas (5). Throughout human evolution, the interactions between the innate immune system and these ancestral microbiota promoted immunoregulation, as they were either part of host physiology (human microbiota), were harmless but inevitably contaminating air, food, and water (environmental microbiota), or were causing severe tissue damage when attacked by the host immune system (e.g., helminthic parasites) (6–8). However, microbial biodiversity and, thus, overall contact with environmental and commensal microorganisms that were present during mammalian evolution and that play a role in setting up regulatory immune pathways, is progressively diminishing in high-income countries, particularly in urban areas (5, 8). The latter is due to sanitation, drinking water treatment, excessive use of antibiotics, changes in diet, feeding of formula milk as a replacement for breast milk, increased caesarean section birth rates, as well as increased time spent within the built environment (6–10). In line with the hypothesis that immunoregulatory capacities of individuals raised in an environment offering a narrow range of microbial exposure are compromised, compared with immunoregulatory capacities of individuals raised in an environment offering a wide range of microbial exposure, we recently showed that young, physically and emotionally healthy, male participants raised during the first 15 years of life in a city with more than 100,000 residents and in the absence of daily contact with pets (urban; *n* = 20) show an increased stress-induced inflammatory response when exposed to the Trier Social Stress Test (TSST) (11), relative to respective participants raised on a farm in the presence of daily contact with farm animals (rural; *n* = 20). In detail, this was indicated by an aggravated stress-induced increase in peripheral blood mononuclear cell (PBMC) counts and plasma interleukin (IL)-6 concentrations, as well as an enhanced Concanavalin A (ConA)-induced IL-6 response from *ex vivo* cultured PBMCs in urban participants with no daily animal contact (8). Importantly, there were no basal immunological differences between the groups before stressor exposure and stress-induced physiological responses also did not differ between the groups (8).

The microbiome data presented in the current study were collected in the identical cohort of participants recruited in our recent study (8) to test whether the increased TSST-induced immune activation in urban participants raised in the absence of daily pet contact, relative to rural participants raised in the presence of daily contact with farm animals, reported in this recent study (8), is accompanied by measureable differences in the composition of the salivary microbiome employing 16S rRNA gene sequencing. This hypothesis is based on recent findings that environment influences the human salivary microbiome, more so than host genetics (12), and the proposal that the oral microbiome may be useful in both the diagnosis and treatment of disease, including inflammatory disease (13, 14).

## Methods

### Recruiting

Recruiting was performed as published recently (8). Briefly, all participants were male, between 20 and 40 years of age, and grew up (until the age of 15) either in a city with more than 100,000 residents and in the absence of daily contact with pets (urban: *n* = 20) or on a farm with daily contact with farm animals (rural: *n* = 20). All participants were physically (i.e., asked whether they suffered from chronic physical disorders) and emotionally healthy (i.e., based on responses to the Structured Clinical Interview for DSM-IV Disorders, SCID-I, administered during telephone screening) and asked to abstain from any kind of drugs (e.g., analgesics, sleep-inducing drugs, dietary supplements), exercise, caffeine, alcohol, and nicotine for a minimum of 3 days before the test day. Furthermore, participants were told to sleep at least 8 h during the night before the experiment and to drink at least 1 l of water on the experimental day itself. The detailed inclusion and exclusion criteria for participants of the current study are reported elsewhere (8). In cases of unforeseen illness, test persons were told to delay the experiment. All experiments were approved by the Ethics Committee of Ulm University and the study is registered at the DRKS (German Clinical Trials Register, ID DRKS00011236). Moreover, a commuting accident insurance policy was installed for participating volunteers. Experimenters were covered by the employer's public liability insurance. All Data and Samples were collected between October 2016 and April 2017. Sociodemographic-, psychometric, physiological and immunological data from all participants of the present study have already been published recently (8).

### Experimental Procedure

The detailed experimental procedures have already been described elsewhere (8). Briefly, sociodemographic features were assessed by questionnaire at the beginning of the experimental procedure. All participants were asked whether they had no animal contact at all, occasional animal contact, or daily animal contact until their 15^th^ birthday, respectively. Importantly, while rural participants (*n* = 20) were only included in the study if they indicated daily contact with farm animals, urban participants (*n* = 20) were included when indicating either no daily animal contact or occasional animal contact until their 15^th^ birthday (i.e., the requirement for enrollment in the study for urban participants was “no daily animal contact”). Five urban participants reported absolutely no animal contact until their 15^th^ birthday. Following verification of emotional and physical health status by validated questionnaires (List of complaints for quantitative analysis of current bodily and general complaints (BL); State-(Trait-)Anxiety-Inventory (STAI-S) Questionnaire), the venous catheter (non-dominant arm), as well as the blood pressure and heart rate monitor (dominant arm) were placed (–60 min time point). Before (–5 min) and after (5, 15, 60, 90, and 120 min) the TSST, different parameters where assessed at each time point. In detail, heart rate and diastolic (D) and systolic (S) blood pressure (BP) were assessed (for calculation of mean arterial pressure (MAP) according to the formula: DBP + (SBP-DBP)/3), blood was drawn in ethylenediaminetetraacetic acid (EDTA) and lithium heparin-coated monovettes for collection of plasma and peripheral blood mononuclear cells (PBMCs), and saliva samples were collected for determination of cortisol concentration and microbiome analysis (for details see next section), respectively. After the 5^th^ blood draw (90 min time point), STAI-S was used again to assess subjective strain induced by the TSST procedure. After the 6^th^ blood draw (120 min) the catheter was removed and mental health status [Hospital Anxiety and Depression Scale - German Version, HADS-D; SCID-I (affective part)], early life (Childhood Experience of Care and Abuse Questionnaire, CECA-Q; Childhood Trauma Questionnaire, CTQ), and perceived life stress (Perceived Stress Scale-4, PSS-4) were assessed using validated questionnaires.

### TSST

Acute psychosocial stress was induced using the TSST. For a detailed description of the testing procedure, see (8).

### Blood Pressure and Heart Rate

BP and heart rate of the participants were determined at time points –5, 5, 15, 60, 90, and 120 min. For details, see (8). As TSST-induced changes in MAP in the original study (8) were most pronounced between time point 1 (baseline; –5 min) and 3 (+15 min), we used the respective area under the curve with respect to the ground (AUC) to compare urban participants who grew up with absolutely no animal contact with urban participants who grew up with occasional animal contact (**Figure 3**) in the present study.

### Blood Draw

Blood was drawn as previously described (8). Briefly, blood (7.5 ml at each time point) was collected from an indwelling venous catheter in the non-dominant arm (inserted at –60 min) at time points –5 (5 min before the start of the TSST), 5 (5 min after termination of the TSST), 15, 60, 90, and 120 min into chilled EDTA-coated monovettes. Additionally, 9 ml of blood were collected at each time point into lithium-heparin-coated monovettes. For details about the processing of the blood samples, see (8).

### PBMC Isolation

For a detailed description of the procedure, see (8). Briefly, nine ml blood were transferred from lithium-heparin-coated monovettes into Leucosep™ tubes (Greiner Bio-One GmbH, Frickenhausen, Germany), which were prepared beforehand with Ficoll^®^ Paque (GE Healthcare Life Sciences, Freiburg, Germany) according to the manufacturer's instructions. The number of viable PBMCs was determined using an automated cell counter. As TSST-induced differences in blood PBMC counts between urban and rural participants in the original study (8) were most pronounced between time point 1 (baseline; –5 min) and 3 (+15 min), we used the respective AUC to compare urban participants who grew up with absolutely no animal contact with urban participants with who grew up with occasional animal contact (**Figure 3**) in the present study.

### Enzyme-Linked Immunosorbent Assay (ELISA)

Plasma samples and supernatants from PBMC stimulations were analyzed using commercially available ELISA kits according to the manufacturers' instructions. In detail, plasma samples were analysed for IL-6 (Quantikine HS ELISA; R&D Systems Europe, Wiesbaden, Germany), and cortisol (IBL International, Hamburg, Germany). As TSST-induced changes in plasma cortisol in the original study (8) were most pronounced between time point 1 (baseline; –5 min) and 3 (+15 min), we used the respective AUC to compare urban participants who grew up with absolutely no animal contact with urban participants who grew up with occasional animal contact (**Figure 3**) in the present study. Accordingly, we calculated AUC between time point 5 (90 min) and 6 (120 min) for plasma IL-6 levels in the current study to compare urban participants who grew up with absolutely no animal contact with urban participants who grew up with occasional animal contact (**Figure 3**), as TSST-induced differences between urban and rural participants in plasma IL-6 levels were only detectable at these late stages.

### Collection and Preparation of Salivary Samples

Salivary samples were collected at the –5, 5, 15, 60, 90, and 120 min time points. For salivary sample collection, a salivette^®^ (Cat. No. 51.1534.500; Sarstedt, Nuernberg, Germany) was used. In detail, each participant was advised to chew a salivette^®^ swab thoroughly for approximately one minute at each time point, then to spit the swab into a sterile tube. Subsequently, samples were centrifuged (1,000 g, 2 min, RT) and stored at –80°C until further processing. For microbiome analysis, salivary samples taken at the –5 min time point were used. 100 µl of saliva was used for the DNA extraction. DNA was extracted using the PowerSoil DNA extraction kit (Cat No. 12888-100 & 12955-4, MoBio Laboratories, Carlsbad, CA, USA) according to the manufacturer's instructions. Marker genes in isolated DNA were PCR-amplified using HotStarTaq Master Mix (Cat No. 203433, Qiagen, Valencia, CA, USA) and the 515 F (5'-GTGCCAGCMGCCGCGGTAA-3')/806 R (5'-GGACTACHVGGGTWTCTAAT-3') primer pair (Integrated DNA Technologies, Coralville, IA, USA) targeting the V4 hypervariable region of the 16S rRNA gene modified with a unique 12-base sequence identifier for each sample and the Illumina adapter, as previously described (15). The thermal cycling program consisted of an initial step at 94°C for 3 min followed by 35 cycles (94°C for 45 sec, 55°C for 1 min, and 72°C for 1.5 min), and a final extension at 72°C for 10 min. PCR reactions were run in duplicate and the products from the duplicate reactions were pooled and visualized on an agarose gel to ensure successful amplification. PCR products were cleaned and normalized using a SequalPrep Normalization Kit (Cat. No. A1051001, ThermoFisher, Waltham, MA, USA) following manufacturer's instructions. The normalized amplicon pool was sequenced on an Illumina MiSeq run using V3 chemistry, 600 cycles, and 2 x 300-bp paired-end sequencing. All sequencing and library preparation were conducted at the University of Colorado Boulder BioFrontiers Next-Gen Sequencing core facility.

### Microbiome Analysis

Microbiome bioinformatics were performed with QIIME2-2019.7 (http://qiime2.org) (16, 17). Briefly, raw sequence data were demultiplexed and quality filtered using the q2‐demux plugin followed by denoising with DADA2 *via* q2‐dada2 (18) to identify all observed amplicon sequence variants (ASVs) [i.e., 100% operational taxonomic units (OTUs)]. All ASVs were aligned with mafft (19) (via q2‐alignment) and used to construct a phylogeny with fasttree2 (20) (via q2‐phylogeny). Alpha‐diversity metrics [observed OTUs, Faith's Phylogenetic Diversity (21), and Shannon diversity index], and beta diversity metrics [weighted UniFrac (22), unweighted UniFrac (23)] were estimated using q2‐diversity after samples were rarefied (i.e., subsampled without replacement). A total of 9,280 sequences per sample were chosen as our rarefaction depth to retain all paired samples, as samples with fewer sequences than the rarefaction depth are excluded from downstream diversity analyses. PCoA plots were generated using the weighted UniFrac distance matrix in R Studio1.2.1335 [RStudio Team (2018). RStudio: Integrated Development for R. RStudio, Inc., Boston, MA URL http://www.rstudio.com/] and Phyloseq package 1.28.0 (24). The differentially abundant features between saliva samples of participants with or without animal contact until their 15^th^ birthday were determined through the analysis of composition of microbiomes (ANCOM) pipeline (25). The microbiome data assessed in the present study are available in the NCBI SRA public repository (accession number: PRJNA606354).

### Statistics

Significant differences in alpha diversity were calculated using the non-parametric Kruskal-Wallis ANOVA on ranks test (26); two-tailed *p* values < 0.05 were considered as statistically significant. Differences in beta diversity were calculated using a generalized UniFrac distance model (27). PERMANOVA pseudo *p* values < 0.05 were considered statistically different. For statistical analysis and graphical illustration of the area under the curve with respect to the ground (AUC) data, the software package Prism (version 8) was used. Raw data sets used for AUC calculation were already corrected for outliers (28) and thus identical to our previously published study (8). Within AUC datasets, Kolmogorov-Smirnov test using Lilliefors' significance was employed to test normal distribution of all acquired data sets. Normally distributed data sets were subsequently analyzed using parametric statistics (Student's *t*-test). Non-normally distributed data sets were analyzed using non-parametric statistics [Mann-Whitney U test (MWU)]. Normally distributed data are presented as bars (mean + SEM). Non-normally distributed data are presented as box plots (median; min, max, 25^th^ and 75^th^ percentile). The two-tailed level of significance was set at *p* < 0.05.

## Results

### Salivary Microbial α- and/or β-Diversity Does Not Differ Between Participants Raised in Rural Areas in the Presence of Daily Contact With Farm Animals Compared With Participants Raised in Urban Areas in the Absence of Daily Animal Contact in Adulthood

**Figure 1:**
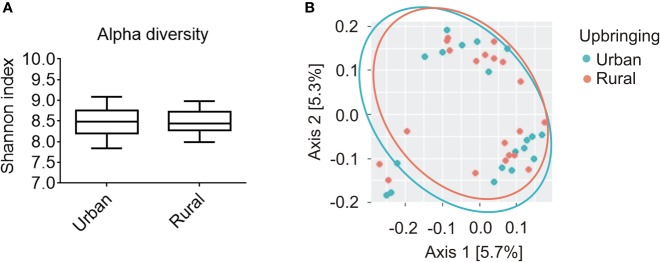
Alpha and beta diversity analysis of the salivary microbiome composition for participants raised in urban areas in the absence of daily animal contact compared to individuals raised in rural areas in the presence of daily contact with farm animals. **(A)** Shannon diversity index representing richness and evenness within samples was not significantly different between urban (*n* = 20) and rural (*n* = 20) populations (Kruskal-Wallis, *p* = 0.935). Solid line represents the median. Lower box indicates 25th, upper box indicates 75th percentile. 10th and 90th percentile are indicated by lower and upper error bar, respectively. **(B)** Weighted UniFrac principal coordinates analysis (PCoA) plot represents beta diversity as phylogenetic distances among samples for both urban (turquoise; *n* = 20) and rural (orange; *n* = 20) populations. PCoA axes 1 and 2 explain 5.7 and 5.3% of the variation, respectively.

Statistical analysis using Kruskal-Wallis-H-Test (KWH) revealed no significant difference in the α-diversity of the salivary microbiome as measured by Shannon diversity index between participants raised in urban areas without daily animal contact with animals vs. participants raised in rural areas in the presence of daily contact with farm animals (*p* = 0.935; **Figure 1A**). Consistent with the results observed with Shannon index, the analysis of Faith's Phylogenetic Diversity and observed OTUs alpha diversity indexes displayed no differences between urban and rural groups (Faith's PD, *p* = 0.999; observed OTUs, *p* = 0.840). There was also no association between salivary β-diversity and the factor urban-rural upbringing (pseudo-*F* = 0.635; *p* = 0.908). The latter is visualized using a weighted UniFrac principal component analysis (PCoA) plot presenting phylogenetic distances among all of the samples (**Figure 1B**).

### Growing Up With Absolutely No Animal Contact Until the 15^th^ Birthday Affects Salivary β-Diversity in Adulthood

**Figure 2:**
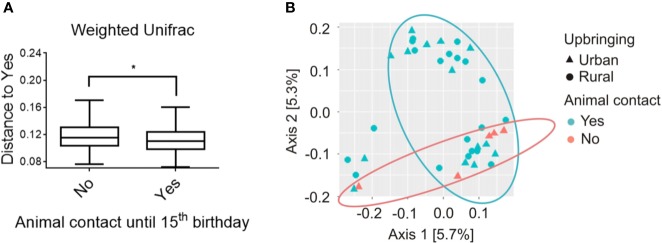
Beta diversity analysis of salivary microbiome composition for participants with absolutely no animal contact compared to participants that were at minimum exposed to occasional animal contact until the 15^th^ birthday, respectively. **(A)** Analysis of beta diversity using weighted-UniFrac distance metrics revealed differences in the microbial community structure of participants with no animal contact at all (red, *n* = 5) compared to participants with at least occasional animal contact until the 15^th^ birthday (blue, *n* = 35), respectively. Distances to subjects with animal contact (Yes = animal contact; No = No animal contact) are represented by a PERMANOVA analysis with a total of 999 permutations, pseudo-*F* = 1.988, *p* = 0.038. Solid line represents the median. Lower box indicates 25th, upper box indicates 75th percentile. 10th and 90th percentile are indicated by lower and upper error bar, respectively. **(B)** Weighted-UniFrac principal coordinates analysis (PCoA) plot shows participants raised in rural areas in the presence of daily contact with farm animals (circles; *n* = 20) and participants raised in urban areas in the absence of daily animal contact (triangles; *n* = 20). Participants were further subdivided into the groups with “absolutely no animal contact until the 15^th^ birthday” (orange, *n* = 5) or “occasional to daily animal contact until the 15^th^ birthday” (turquoise, *n* = 35). PCoA axes 1 and 2 explain 5.7 and 5.3% of the variation, respectively. *p < 0.05.

The microbial β-diversity in “urban” participants raised with absolutely no animal contact until the 15^th^ birthday (*n* = 5) was significantly increased compared with participants reporting either occasional (*n* = 15) or daily (*n* = 20) animal contact during upbringing. This is indicated by a PERMANOVA analysis (pseudo-*F* = 1.988; *p* = 0.038; **Figure 2A**) calculating the distances of each sample to all samples with a minimum of “occasional” animal contact. The latter is further visualized by a weighted-UniFrac PCoA Plot showing the calculated distances between all samples assessed (**Figure 2B**).

### Urban Participants Growing Up With Absolutely No Animal Contact Showed an Increased TSST-Induced Inflammatory Response Compared With Urban Participants Growing Up With Occasional Animal Contact

**Figure 3:**
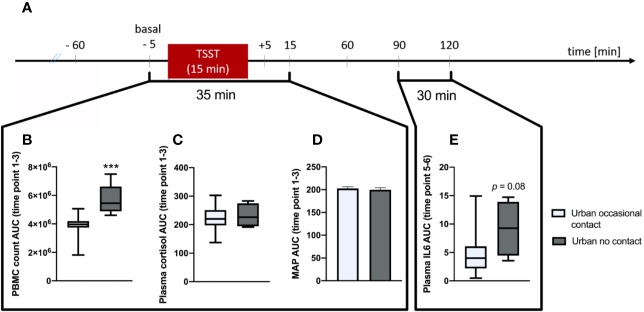
Analysis of Trier Social Stress Test (TSST)-induced alterations on immunological and physiological parameters between urban participants growing up with no animal contact at all vs. urban participants growing up with “occasional” animal contact until the 15^th^ birthday, respectively. **(A)** Timeline of the experimental procedure. At –60 min time point, the venous catheter as well as the blood pressure and heart rate monitor were placed. Before (–5 min) and after (5, 15, 60, 90, and 120 min) the TSST, different immunological and physiological parameters where assessed. **(B)** Area under the curve with respect to ground (AUC) analysis indicates that urban participants growing up with no animal contact at all (*n* = 5) vs. urban subjects growing up with “occasional” animal contact until the 15^th^ birthday (*n* = 15), respectively showed significantly higher counts of plasma peripheral blood mononuclear cells (PBMCs) between time point 1 and 3. This effect was also by trend visible in plasma interleukin (IL)-6 levels between time point 5 and 6 **(E)**. There were no significant differences between both groups in the AUC between time point 1 and 3 of plasma cortisol levels **(C)** and the mean arterial pressure (MAP; **D**), respectively. Normally distributed data are presented as bars (mean + SEM). Non-normally distributed data are presented as box plots (Median, min, max, 25^th^ and 75^th^ percentile) ***p < 0.001.

Statistical analysis revealed that urban participants reporting absolutely no animal contact during their first 15 years of life showed a significantly greater stress-induced increase in blood PBMC counts between time point 1 (baseline; –5 min) and 3 (+15 min) (AUC; *p* < 0.001; MWU; **Figures 3A, B**) than did respective urban participants reporting occasional animal contact. A comparable effect was also by trend detectable in plasma IL-6 levels between time point 5 (+90 min) and 6 (+120 min) (AUC; *p* = 0.08; MWU, **Figures 3A, E**). No significant differences between these two groups were found in plasma cortisol levels (AUC; **Figures 3A, C**) and MAP (AUC; **Figures 3A, D**), both assessed between time point 1 and 3.

## Discussion

In the present study, we showed that urban upbringing in the absence of pets compared to rural upbringing in the presence of farm animals is not associated with specific patterns in the α- or β-diversity of the salivary microbiome. In contrast, we were able to reveal that the salivary microbial β-diversity is significantly different in individuals with no animal contact at all compared to all other participants with at least occasional animal contact until the age of 15, respectively—a finding that was further associated with a more pronounced immune activation within the group of urban participants following acute psychosocial stressor exposure induced by the TSST.

In line with the hypothesis that an overreactive immune system is causally involved in the pathogenesis (29, 30) and increased prevalence (3–5) of stress-associated disorders in urban vs. rural environments, we recently showed that healthy male individuals raised in urban areas without daily animal contact vs. individuals raised in rural areas in the presence of daily contact with farm animals respond with increased systemic immune activation towards the TSST (8), a standardized laboratory stressor in humans (11). Given that a modern urban lifestyle is associated with less contact to biodiversity (31), and that a reduced exposure to microbial antigens (32, 33) and immunoregulatory “Old Friends” microorganisms (6, 7, 34, 35), especially during early life (36), promotes development of inflammatory disorders later in life (37, 38), a role of the human microbiome in the accumulation (4, 39) of stress-associated disorders in urban vs. rural environments is very likely. Surprisingly and against our hypothesis, neither α- nor β-diversity measures were significantly different between the two experimental groups in the present study, suggesting the composition of the salivary microbiome in adulthood is not a reliable marker of the environment that an individual was raised in. A possible reason for this finding might be that differences in the salivary microbial composition between “urban” and “rural” individuals at a young age are just not surviving into an individual's adulthood. In support of this hypothesis, the mouth cavity is a rapidly changing environment for bacteria, affected by diet and sanitation (40), the currently prevailing environment, and host age (12, 41). In line with this possibility, C-section-induced differences in gut microbial composition are only present throughout the neonatal period, but largely disappear within the first years (42).

As the overall extent of animal contact during upbringing has been shown recently to affect the composition of the human gut microbiome (43, 44), we investigated in a next step if the salivary microbial diversity in adulthood is different between participants who reported absolutely no contact with pets compared to participants who reported occasional or daily contact with pets or farm animals. Notably, whereas the main criterion for rural participants (*n* = 20) to be included into the study was to have had daily contact with farm animals until the age of 15, urban participants (*n* = 20) were included when indicating either no animal contact at all or occasional animal contact until their 15^th^ birthday. Interestingly, and in support of the above-reported hypothesis, comparing participants with no animal contact at all (*n* = 5; exclusively urban participants) to participants exposed to occasional (*n* = 15; urban participants) or daily (*n* = 20; rural participants) animal contact until the age of 15 (overall *n* = 35), respectively, revealed a significant difference in β-diversity of the salivary microbiome, indicating that, in contrast to the environment that an individual is brought up in, the extent of animal contact during childhood potentially has long-lasting effects on an individual's salivary microbiome. Our finding that microbial β-diversity was significantly higher in urban participants reporting absolutely no animal contact until the age of 15 years compared to all other participants reporting either daily contact to farm animals (rural) or occasional pet contact (urban) is in line with the fact that Westernization has been consistently associated with lower α-diversity but higher β-diversity (9). As microbiome alterations associated with Westernization are hypothesized to be mainly driven by dispersal limitation in combination with high inter-individual differences in selective environments, our findings suggest that a reduction in animal contact might be at least one factor contributing to the overall dispersal limitation in Western societies and the consequently increased risk to develop non-communicable diseases (9).

Of particular interest in the context of these results is a study showing that living on single-family dairy farms with regular contact with farm animals in Amish farm children goes along with a lower asthma and allergy risk and innate immune system activation compared to living on highly industrialized farms with little contact with farm animals in Hutterite farm children (38). In accordance with these findings, other studies revealed that early exposure to both pets and farm animals reduces the risk of childhood asthma and other inflammatory disorders (45, 46). Strikingly, comparing TSST-induced changes in blood PBMC counts at the initial phase of stress [urban vs. rural differences were most pronounced between time point 1–3 (8)] and plasma IL-6 levels at the late phase [urban vs. rural differences were most pronounced between time point 5–6 (8)] between urban individuals with absolutely no animal contact vs. occasional animal contact during upbringing, revealed that the former showed a significantly greater stress-induced increase in blood PBMC counts and by trend increased plasma IL-6 levels, both quantified as area under the curve between respective time points. In contrast to the effects on stress-induced immune activation and in contrast to the hypothesis that increased hypothalamic–pituitary–adrenal (HPA) axis activity is associated with the development of stress-related psychiatric disorders (47), the overall animal contact during upbringing did not affect TSST-reactivity of the HPA axis and the cardiovascular system. This was indicated by comparable stress-induced increases in plasma cortisol levels and mean arterial pressure at the initial phase after TSST between urban participants raised with no vs. occasional animal contact, respectively. However, given that these parameters were also comparable between rural vs. urban participants raised in the presence or absence of animals, respectively (8), an exaggerated HPA axis and sympathetic nervous system activity seem to only play a minor role in mediating the increased prevalence of stress-associated disorders in urban vs. rural environments.

Nevertheless, our study has some limitations that have to be taken into consideration. One limitation in the current study is that the sample size in the urban group raised without any pet contact is small, clearly indicating the rather preliminary character of our findings and the need for confirmation in adequately powered future studies. Of note, statistical analysis employing partial least squares discriminant analysis (PLSDA) as a supervised learning approach (48, 49) did not reveal that the salivary microbiome was predictive of urban status with or without animal contact. However, as discerning subtle variations in community configurations along the pet-contact/urban or rural living categories would add important mechanistic knowledge on the involved bacterial species mediating these beneficial long-term effects of animal contact, these type of analyses should be repeated employing larger cohorts of participants. Additional limitations are that we did not take into account possible differences in participants' mode of delivery at birth, antibiotic usage during the first years of life, feeding of formula milk as a replacement for breast milk, or exposure to kindergarten, which are all known to affect the composition of the microbiome (38, 50–52).

Together with our previous work (8), the results of the present study suggest that the complete absence of animal contact in early life during urban upbringing promotes life-long differences in the composition of the salivary microbiome and potentiates the negative consequences of urban vs. rural upbringing on stress-induced immune activation. As urban participants in the present study reporting animal contact were only included if this contact was occasional and not daily, we are convinced that future studies comparing urban participants raised in the absolute absence vs. daily presence of pets will reveal even more prominent differences in TSST-induced immune activation between the groups.

## Data Availability Statement

The datasets generated for this study are available on request to the corresponding author.

## Ethics Statement

The studies involving human participants were reviewed and approved by Ethics Committee of Ulm University. The participants provided their written informed consent to participate in this study.

## Author Contributions

GR, CL, HG, CW, and SR designed the study. DL, CZ, TB, SH, MJ, NR, CS, JH, CW, and SR performed experiments. DL, CZ, CL, and SR analyzed data. DL, CZ, CL, and SR wrote the manuscript.

## Conflict of Interest

The authors declare that the research was conducted in the absence of any commercial or financial relationships that could be construed as a potential conflict of interest.
